# Analysis of the Spatial Distribution Characteristics of Urban Resilience and Its Influencing Factors: A Case Study of 56 Cities in China

**DOI:** 10.3390/ijerph16224442

**Published:** 2019-11-12

**Authors:** Maomao Zhang, Weigang Chen, Kui Cai, Xin Gao, Xuesong Zhang, Jinxiang Liu, Zhiyuan Wang, Deshou Li

**Affiliations:** 1 Hubei Province Key Laboratory for Geographical Process Analysis and Simulation, Wuhan 430079, China; 2 College of Urban and Environmental Sciences, Central China Normal University, Wuhan 430079, China; 3 School of Architecture, University of South China, Hengyang 421001, China; 4 Institute of Geological Survey, Hebei GEO University, Shijiazhuang 050031, China; 5 Business School, Hohai University, Nanjing 211100, China; 6 School of Civil Engineering, University of South China, Hengyang 421001, China

**Keywords:** urban resilience, spatial distribution, influencing factor, spatial regression model, 56 cities

## Abstract

The healthy development of the city has received widespread attention in the world, and urban resilience is an important issue in the study of urban development. In order to better provide a useful reference for urban resilience and urban health development, this paper takes 56 cities in China as the research object, and selects 29 indicators from urban infrastructure, economy, ecology and society. The combination weight method, exploratory spatial data analysis (ESDA) and spatial measurement model are used to explore the spatial distribution of urban resilience and its influencing factors. From 2006 to 2017, the urban resilience of prefecture-level cities in the four provinces showed a wave-like rise. During the study period, the urban resilience values, measured as Moran’s Is, were greater than 0.3300, showing a significantly positive correlation in regard to their spatial distribution. Regarding the local spatial correlation, the urban resilience of the study area had spatial agglomeration characteristics within the province, with a significant distribution of “cold hot spots” in the spatial distribution. From the perspective of the factors that affected urban resilience, the proportion of the actual use of foreign capital in GDP and carbon emissions per 10,000 CNY of GDP had a negative impact and GDP per square kilometer, the proportion of urban pension insurance coverage, the proportion of the population with higher education, and expenditure to maintain and build cities had a positive impact. The development strategy of urban resilience must be combined with the actual situation of the region, and the rational resilience performance evaluation system and the top-level design of urban resilience improvement should be formulated to comprehensively improve urban resilience.

## 1. Introduction

The city is a complex system of the integration of society, economy, ecology and infrastructure and their roles are gaining increasing attention worldwide [1,2,3]. Particularly in China, where urbanization is placed strategically, the role played by cities has become more critical and obvious [4,5]. Since China’s reform and opening up, the level of urbanization has increased by nearly 1% every year [6]. Urban space expansion is the main feature of China’s urbanization process [7,8]. Since the beginning of the 21st century, as the pace of urban development in China has accelerated [9,10], urban activities have become highly concentrated, and spatial imbalances have repeatedly appeared. Coupled with the intrusion of natural disasters, the city is suffering and has endured serious disasters. According to relevant statistics, the number of deaths caused by sudden natural events such as natural disasters and public safety in China has exceeded 200,000. At the same time, the number of people affected by disasters has reached 1.5–350 million, and the economic losses have exceeded 650 billion yuan [11]. Such huge losses have brought serious negative effects and huge threats to urban development, have undermined the city’s development system and have reduced the quality of urban operations [2,12]. However, when cities solve urban problems and face the impact of different disasters, the stiffness, intensity, redundancy and resilience of the city will be different, and the significant difference in resilience within the urban system is the main underlying cause [13,14]. Urban development concepts such as “urban sustainable development”, “ecological green city” and “healthy city” are gradually being accepted and valued, and these are placed on the healthy development agenda of the city. This paper considered 56 Chinese cities as an example to study urban resilience and explore its spatial distribution characteristics and influencing factors, which have important reference significance to enhance and improve urban resilience and promote urban health science development.

The resilience city originated from the Latin word “resilio” [15], and its original meaning was to return to the original state. In the 1970s, the concept of resilience began to be introduced into the field of ecological research. The Canadian ecologist Holling used the term “resilient” to describe the characteristics of ecosystems, and it gradually became the basic factor for ecosystem construction [16]. For ecologists, the resilience concept focuses on the strong adaptability of the ecosystem in the face of man-made or natural disasters and the ability to repair after a disaster. With the deepening of people’s understanding of the concept of resilience, the connotation of resilience has been continuously developed. At the same time, scholars have begun to combine resilience with urban studies and open up new horizons in urban studies [17]. Subsequently, the Panarchy model, adaptive cycle and multi-scale nested adaptive cycle model to study the dynamics of ecological resilience were proposed [18]. Some scholars have believed that urban resilience reflects the city’s adaptability and self-repair ability in the face of unpredictable disasters [19,20]. Additionally, a few scholars have regarded resilience as a process and consider it a continuous learning and decision-making ability to cope with various disasters that occur at any time, and a crisis management strategy [21,22]. However, economists have suggested that urban resilience is the ability of the urban economy to protect itself and reduce losses, and it is the embodiment of urban flexibility and vitality after disasters [23,24]. More research suggests that resilience should be systematically combined with urban planning to explain the evolution of resilience from the perspective of urban construction. Therefore, more research has evaluated urban resilience from the perspectives of demographics, social systems and community organizations and has made recommendations for its sustainable development [25,26].

With the continuous development of China’s new urbanization, the issue of urban resilience has attracted increasing attention from scholars. In recent years, Chinese scholars have also carried out many exploratory studies on urban resilience, many of which have focused on urban resilience assessments and urban self-recovery capabilities in a few megacities and economically developed urban agglomerations on the eastern coast of China [27,28]. A few scholars have explored urban ecological resilience from the perspective of time and space evolution [29,30]. Some scholars have also quantitatively evaluated and explored the resilience of urban infrastructure from green infrastructure, water supply systems, drainage systems and transportation track networks [31,32]. At the same time, many studies have reported on the connotation and characteristics of urban resilience from the aspects of physical, social and informational three-dimensional systems [33]. They focused more on urban resilience management and promoted the rational allocation of limited resources to urban subsystems to enhance urban resilience. Additionally, some scholars have studied the construction of resilient cities in developed countries, summarized the construction of resilient cities in developed countries from the perspective of policy process, and provided important references for urban planning and construction in China [34]. In general, urban resilience mainly depends on the coordination of multiple subsystems in the city, that is, when the city is impacted and disturbed by its own environment and the external environment (natural and man-made disasters, diseases, resource depletion, etc.), the city’s organization, community, infrastructure, economy, society and ecology and other systems will show the ability to respond and defend in a timely manner and quickly restore the original state [35,36,37,38,39,40].

From the existing literature, the research scale mainly focuses on countries, provinces and a single large-scale city. However, there are insufficient horizontal comparison studies between multiple cities. Moreover, many studies have limited urban resilience assessment to only one subsystem of the city. However, because the city is a complex multi-element system, this simple study is insufficient to assess the overall resilience of the city. Additionally, most of the research comprised only a simple calculation and evaluation of urban resilience, but few studies exist on its temporal and spatial distribution characteristics and impact factors. Thus, this paper considered 56 prefecture-level cities in Shaanxi, Henan, Anhui and Jiangsu Provinces as research objects, and used the combined weight method and multi-index comprehensive evaluation method, together with the spatial analysis of ArcGIS 10.2 (ESRI, 380 New York Street, Redlands, CA, USA) and spatial regression model, to study urban resilience and its influencing factors. The main advantages of this paper are as follows:

(1) A typical study area was selected: 56 prefecture-level cities in four provinces, including the western, central, and eastern regions with different degrees of development in China, that can more comprehensively reflect the overall urban resilience of China were selected; (2) the evaluation index system is relatively complete: to evaluate urban resilience more comprehensively, this paper adds the resilience evaluation index of the urban ecosystem and combines the three aspects of urban society, economy and infrastructure, finally establishing the urban resilience evaluation index system including 29 evaluation indicators; (3) comprehensive content of the research: this paper combines the multi-angle quantitative evaluation and the spatial visualization method to comprehensively analyze the urban resilience and dynamic evolution characteristics of the research area from 2006 to 2017. Furthermore, to provide a reference for urban planning and sustainable development, the spatial regression model was introduced to explore the influencing factors of urban resilience.

## 2. Research Area and Data Sources

### 2.1. Research Area

**Figure 1:**
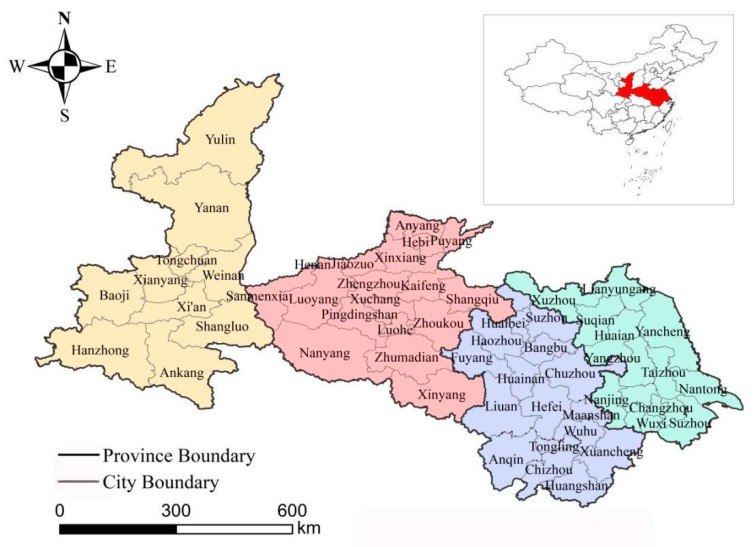
Location of the study area.

This study considers the four Chinese provinces of Shaanxi, Henan, Anhui and Jiangsu as research areas, which span 105°29′–121°57′ E, 29°41′–39°35′ N, with a total area of 6.20 million km^2^, accounting for 6.44% of China’s total land area. Shaanxi Province is one of the “gateway” provinces in Western China, and it is located in an important position connecting the Eastern and Central China regions and the northwest and southwest regions [41]. Henan Province is the most populous province and a major grain producer, and it is an important transportation hub connecting the east and west regions and north and south regions in China [42]. Anhui Province is located in the East of China and spans the Yangtze River and Huaihe River [43]. Anhui Province is an important agricultural production, energy, raw material and processing and manufacturing base in China. Jiangsu Province is located in the eastern coastal areas of mainland China, and it is one of the most economically active provinces in Eastern China [44]. The Yangtze River Delta urban agglomeration formed by Anhui Province, Jiangsu Province, Shanghai and Zhejiang Province has become one of the six world-class urban agglomerations in the world. This study mainly used prefecture-level cities as the basic research unit, involving 56 prefecture-level cities, as shown in Figure 1.

### 2.2. Data Sources and Pre-Processing

The main sources of data in this paper are the China City Statistical Yearbook 2006–2017, Jiangsu Statistical Yearbook 2006–2017, Henan Statistical Yearbook 2006–2017, Anhui Statistical Yearbook 2006–2017 and Shaanxi Statistical Yearbook 2006–2017. Furthermore, we referred to the provincial statistical bulletin for the 2006–2017 study area. The indicators are mainly based on the main city of the prefecture-level city (including the municipal district) as the statistical caliber. Some indicators are replaced by the municipal district, and the individual missing values are complemented by the interpolation method in SPSS.22 (International Business Machines Corporation (IBM): Armonk, NY, USA) software. To eliminate the difference in the dimensions, this paper used the method of the min-max normalization to standardize the raw data [39]; the specific equations are as follows:

- For the larger and better positive indicators, the standardization equation is as follows:
  (1) $$Y_{ij}=\frac{X_{ij}-\max(X_{ij})}{\max(X_{ij})-\min(X_{ij})}$$
- For the negative indicator of the smaller and better, the standardization equation is as follows:
  (2) $$Y_{ij}=\frac{\max(X_{ij})-X_{ij}}{\max(X_{ij})-\min(X_{ij})}$$

In Equations (1) and (2), $\max(X_{ij})$ and $\min(X_{ij})$ represent the maximum and minimum values of the index of the jth indicator of the ith city, respectively, and $Y_{ij}$ represents the value of the jth indicator of the ith city after normalization.

## 3. Methods

### 3.1. Index of Urban Resilience Assessment

Based on the current analysis of the connotation of resilient cities and availability of data [45,46,47,48,49,50,51], we considered the urban infrastructure, economy, ecological society and integration of highly complex coupled systems [52]. To better reflect the spatial and temporal changes in urban resilience in the study area, as well as ensure the reliability and effectiveness of urban resilience measurements, this paper adheres to the systemic, scientific, objective and feasibility principles of the indicator selection process and establishes a comprehensive index system for urban resilience evaluation from four aspects: urban infrastructure, economy, ecology and society [51,53].

The risk of urban infrastructure is mainly manifested in the large-scale agglomeration of the population, which has exerted tremendous pressure on urban electricity, road networks and telecommunications, resulting in obvious vulnerability in the face of accidental natural disasters. In this regard, seven indexes were selected—number of internet users per 100 people, number of health care beds per 10,000 people, number of public transportation vehicles per 10,000 people, per capita area of paved roads in the city, per capita annual electricity consumption, per capita postal expenditure and number of mobile phones per 100 people—to evaluate the resilience of urban infrastructure to resist risks. Urban economic resilience is mainly reflected in the economic stability of the city when faced with the impact of uncertain economic factors. Among them, the eight indicators, the fiscal deficit rate, proportion of private and individual employment in urban areas to the number of employed people in the city, proportion of GDP increased by the tertiary industry, financial interrelations ratio, industrial structure diversification index, proportion of financial expenditure on science and technology, per capita GDP and per capita retail sales amount of consumer goods, have obvious influence on the economic resilience of the city. The ecological risks in the process of urban development are mainly reflected in the increase in the urban impervious floor area, reduction of the green space landscape and excessive discharge of pollutants, which increase the risk of energy flow interruption and ecosystem load. Therefore, this paper selected seven indicators, the electricity consumption per 10,000 Chinese Yuan (CNY) of GDP, volume of sulfur dioxide emissions, volume of industrial waste water discharged, volume of industrial soot (dust) emissions, green coverage rate in urban constructed areas, per capita area of parks and green land and ratio of industrial solid wastes comprehensively utilized, to evaluate urban ecological resilience. The social resilience of the city is mainly reflected in the ability and development potential of the city in the event of short-term or cumulative shocks. Thus, seven indicators are selected, the number of doctors per 10,000 people, proportion of employees in public administration and social organizations, proportion of education and financial expenditure, collections of public libraries per 100 persons, per capita household deposit balance, proportion of unemployment in urban areas and average wage of employed staff and workers, to evaluate urban social resilience. The specific evaluation indicators are shown in Table 1.

### 3.2. Determination of Indicator Weights

The traditional methods to determine the weight of the target attribute (such as the Delphi method and expert system method) have strong subjectivity, and it is difficult to accurately describe the attribute weight of the indicator [53,54,55]. The entropy weight method is mostly used for static methods, but it easily ignores the time dynamic effects of the indicators, and the time series weights can effectively compensate for the shortcomings. Therefore, to improve the rationality and scientificity of index weights, this paper used the assignment method of the entropy weight method combined with time series weights.

#### 3.2.1. Entropy Weight Method

The entropy method can not only reflect the effect value of index information, but also overcome the information overlap between indicators. Therefore, it is widely used in social economic research fields [56,57]. The equations are as follows:

- Calculate the proportion of the ith city indicator value under the jth indicator:
  (3) $$P_{ij}=\frac{Y_{ij}}{\sum_{i=1}^{m}Y_{ij}}$$
- Calculate the entropy value $e_{j}$ of the jth indicator of the tth year:
  (4) $$e_{j}=-k\sum P_{ij}\ln P_{ij};\;\;\;k=1/\ln m$$
- Calculate the difference coefficient $g_{j}$ of the evaluation index j:
  (5) $$g_{j}=1-e_{j}$$
- Calculate the weight $W_{jt}$ of the indicator j of the tth year:
  (6) $$W_{jt}=g_{j}/\sum g_{j}$$

#### 3.2.2. Weight of Time Series

The urbanization rate is an important manifestation of the comprehensive ability of the city’s social productivity development, scientific and technological progress, improvement of people’s living standards and improvement of infrastructure [58,59,60]. The volatility of the urbanization rate has a crucial impact on the city’s resilience; thus, it can be introduced to reduce the impact of time dynamic factors on the comprehensive assessment of urban resilience. By calculating the growth rate of the total urbanization rate of the study area from 2006 to 2017 and annual urbanization rate, we can calculate the urbanization contribution of the annual growth rate and then take the proportion of the annual urbanization growth contribution as the weight of its time series [45]. The greater the weight value is, the greater the impact on urban resilience is, and conversely, the smaller the weight value, the smaller the impact on urban resilience. The equations are as follows:

(7) $${Con}_{t}=\frac{{gro}_{t}}{Gro}$$

(8) $$W_{t}=\frac{{Con}_{t}}{\sum {Con}_{t}}$$

In Equations (7) and (8), ${Con}_{t}$ represents the contribution rate of urbanization growth in the ith year, ${gro}_{t}$ represents the growth rate of urbanization rate in the study area of the jth year, Gro represents the growth rate of the total urbanization rate in the study area from 2006 to 2017, and $W_{t}$ is the weight of the time series.

#### 3.2.3. Determination of Comprehensive Weights

The final weight value of the evaluation index can be calculated by combining the entropy weight method with the weight of the time series, and the final comprehensive weight is shown in Table 1. The equation is as follows:

(9) $$W_{j}=\sum W_{jt}\cdot W_{t}$$

where $W_{jt}$ represents the weight value obtained by the entropy weight method for the indicator j in the tth year, $W_{t}$ represents the time series weight value of the tth year, and $W_{j}$ represents the final weight value of the j indicator.

### 3.3. Urban Resilience Measurement Model

Urban resilience is calculated using the multi-index weighted summation method of urban infrastructure resilience, urban economic resilience, urban ecological resilience and urban social resilience. The equation is as follows:

(10) $$URI=\sum \left(UIR\cdot W_{j}+UE_{1}R\cdot W_{j}+UE_{2}R\cdot W_{j}+USR\cdot W_{j}\right)$$

where the UIR, $UE_{1}R$, $UE_{2}R$ and USR represent the evaluation indicators of urban infrastructure, urban economic resilience, urban ecological resilience, and urban social resilience, respectively, and URI represents the comprehensive resilience of the city.

### 3.4. Exploratory Spatial Data Analysis (ESDA)

The association characteristics between urban resilience spaces are characterized by the ESDA, which can effectively reveal the spatial interaction mechanism of the research objects [61]. The ESDA can be divided into global and local spatial autocorrelation analysis, in which the global index measures the overall trend of spatial correlation of spatial neighboring cell unit values in the whole research area [62], and the equations are as follows:

(11) $$Moran’I=\frac{\sum_{i=1}^{n}\sum_{j=1}^{n}W_{ij}(X_{i}-\bar{X})(X_{i}-\bar{D})X}{S^{2}\sum_{i=1}^{n}\sum_{j=1}^{n}W_{ij}}$$

(12) $$S^{2}=\frac{1}{n}\sum_{i=1}^{n}\left(X_{i}-\bar{X}\right)$$

In Equations (11) and (12), $\bar{X}$ represents the average value of the attribute value, $X_{i}$ of a certain element of i, and $W_{ij}$ is the spatial weight matrix. The global Moran index range is [−1, 1], and the Moran index value at a given level of significance is positive (negative), indicating the spatial agglomeration (differentiation) of the observed object in the whole universe. The local agglomeration characteristics of the observed objects can be further characterized by local indicators of spatial association (LISA) diagrams, which are divided into four types: high-high (H-H), high-low (H-L), low-low (L-L) and low-high (L-H). The “H-H” type represents that the high-valued region is surrounded by high-value neighbors, the “H-L” type represents a high-valued area surrounded by low-value neighbors, the “L-L” type represents that the low-value region is surrounded by low-value neighbors, and the “L-H” type represents that the low-value region is surrounded by high-value neighbors [63,64].

### 3.5. Spatial Regression Model

Because urban resilience is easily affected by its spatial location, the observations of the research unit are not completely independent of each other, and there is often strong spatial dependence (that is, the observation value of a location depends on the neighboring observations of the neighboring regions), the traditional ordinary least squares model (OLS) will have a large bias [65]. Therefore, this paper uses a spatial regression model to measure the spatial spillover effect of urban resilience. The common spatial regression models include the spatial lag model (SLM) and spatial error model (SEM) [66].

The SLM mainly discusses whether the variable has a diffusion effect (i.e., spatial spillover effect) in a certain area [62], which is an extension of the OLS model, considering the case where the dependent variable observations on spatial unit $A_{i}$ (*i* = 1, 2,…, *n*) depend on the observations of its neighboring region $A_{j}(j\neq 1)$. The equation is as follows:

(13) $$y=\rho \sum_{j=1}^{n}W_{ij}+\sum_{q=1}^{Q}X_{iq}\beta_{q}+\varepsilon_{i}$$

where y represents the interpreted variable, X represents the explanatory variable, $W_{ij}$ represents the $W_{n\times n}$ element of the spatial weight matrix (i,j)th, ε represents the random error vector, and the parameter ρ represents the spatial regression coefficient, which reflects the degree to which the spatial neighboring unit interprets the interpreted variable, and β reflects the influence of the explanatory variable X on the interpreted variable y.

The SEM is a method to address the spatial dependence of error terms. It can be regarded as a combination of standard regression models and spatial autoregressive models of error terms. The most commonly used is the first-order spatial autoregressive model of error [62,67], whose equations is as follows:

(14) $$y=\sum_{q=1}^{Q}X_{iq}\beta_{q}+\varepsilon_{i}$$

(15) $$\varepsilon_{i}=\lambda \sum_{j=1}^{n}W_{ij}\varepsilon_{i}+\mu_{i}$$

where λ represents the autoregressive parameter, which measures the spatial dependence of the disturbance error term, and $\mu_{i}$ represents the random error term. The meanings of the other relevant variables of equations are the same as those in Equation (13).

Because of the endogeneity of independent variables in the spatial regression model, in order to ensure the unbiasedness and validity of the regression results estimation. Anselin proposed using the maximum likelihood method to estimate the SLM and SEM [68]. In the model selection of SLM and SEM, Lagrange multipliers (LM-lag and LM-error) and their robust LM diagnostics (robust LM-lag and robust LM-error) are used to determine which model is more suitable. Anselin proposed which model is more in line with the actual discriminant criterion [69]—that is, if LM-lag is more significant than LM-error in statistics, and robust LM-lag is significant and robust LM-error is not significant, then the SLM model is more appropriate; otherwise, the SEM model is more suitable [70]. If the advantages and disadvantages of the SLM, SEM, and OLS models cannot be accurately determined by the LM-lag and LM-error statistics, other test indicators such as goodness of fit (R^2^), log likelihood value, Akaike information criterion (AIC) and Schwarz criterion (SC) can be used. In general, the larger the goodness of fit and log-likelihood function are and the smaller the Akaike information criterion and Schwarz criterion are, the better the model fitting effect is [71].

## 4. Results and Analysis

### 4.1. Comprehensive Evaluation of Urban Resilience

Using the comprehensive calculation of the data from Formulas (1)~(10), the basic level of resilience from 2006 to 2017 in Shaanxi, Anhui, Henan and Jiangsu provinces can be obtained (Table 2). Table 2 shows that, during the study period, the urban resilience values of prefecture-level cities in the four provinces showed a wave-like rise, but most of the values were approximately 0.23~0.40, and the absolute value was relatively small, indicating that there is still large room for improvement in urban resilience in the study area. There were obvious regional differences and imbalances in China’s urban resilience, and the urban resilience in Jiangsu Province in the east was much higher than that in the other three provinces in Central and Western China. The cause may be that Jiangsu Province is located in the southeast coastal area where China’s economy is rapidly rising, and the city’s infrastructure improvement, economic structure, social development level and ecological control measures are better than those of the central and western regions, indicating that Jiangsu Province can respond quickly and better during corresponding shocks, and its adaptive ability is stronger than that of cities in the central and western regions.

Compared with 2006, the overall urban resilience of the four provinces increased in 2017, and the degree of increase followed the order Anhui > Henan > Shaanxi > Jiangsu, mainly due to the implementation of the “Rise of Central China” and “Western Development” strategies during the study period that were related to the support of the country’s tilting fiscal policy. During the study period, the urban resilience of the four provinces showed a different degree of decline in 2008, reflecting that the 2008 global financial crisis had a negative effect on urban resilience.

### 4.2. Spatial Autocorrelation Analysis of Urban Resilience

We used ArcGIS 10.2 software to calculate the global spatial autocorrelation Moran index of urban resilience from 2006 to 2017 (Table 3). Table 3 shows that the urban resilience values of prefecture-level cities in each study area were greater than 0.3300 and *p* values were all 0.01, indicating that it passed the 1% significance level test. From 2006 to 2017, the Moran index has a clear trend of volatility as a whole, indicating a significant positive correlation in the spatial distribution of urban resilience in the study area, and the agglomeration characteristics are significant. Furthermore, the spatial autocorrelation of urban resilience in the study area on the time scale was further strengthened.

**Figure 2:**
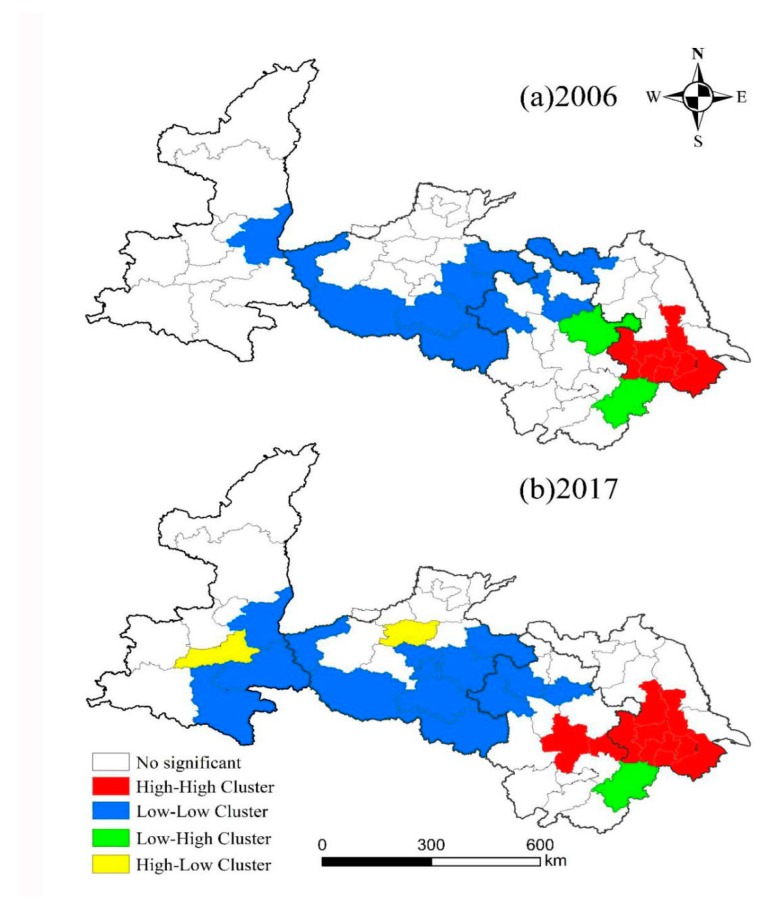
The LISA agglomeration of urban resilience in 2006 and 2017.

Figure 2 further shows the spatial correlation of urban resilience, and the urban resilience of the study area has spatial agglomeration characteristics within the province. The spatial agglomeration characteristics of urban resilience in the eastern, central and western parts of China are obvious, and a significant distribution of “cold hot spots” exists in the spatial distribution. In 2006, the urban resilience “H-H” cluster (high-efficiency type) was mainly distributed in Nanjing, Zhenjiang, Changzhou, Wuxi, Suzhou and Taizhou in Jiangsu Province. The “L-L” agglomeration area (inefficient type) is mainly distributed in Weinan City of Shaanxi Province, the southern and eastern parts of Henan, including Sanmenxia City, Nanyang City, Zhumadian City, Xinyang City, Zhoukou City and Shangqiu City. Fuyang City, Huaibei City, and Handan City in Anhui Province and Xuzhou City in the northwest of Jiangsu Province also belong to this area. The “L-H” agglomeration area (hollow type) is mainly concentrated in the city of Zhangzhou in the eastern part of Anhui Province and Xuancheng City in the southeast. At the end of 2017, the scope of the “H-H” agglomeration area was further expanded, and the expansion area was mainly concentrated in Yangzhou City of Jiangsu Province and Hefei City and Ma’anshan City of Anhui Province. The “L-L” agglomeration area also showed large changes. Among them, the areas with expansion were mainly concentrated in Ankang City and Shangluo City of Shaanxi Province, Luohe City and Xuchang City of Henan Province, and the city of Weizhou in Anhui Province. At the same time, the areas where shrinkage occurred in some areas were mainly distributed in Xuzhou City, Jiangsu Province. The range of the “L-H” agglomeration area was reduced, only appearing in Xuancheng City, Anhui Province. The “H-L” agglomeration area (polarized type) was mainly distributed in Xi’an City of Shaanxi Province and Zhengzhou City of Henan Province.

### 4.3. Spatial Regression Analysis of Influencing Factors of Urban Resilience

To gain a deeper understanding of the influencing factors of urban resilience and provide more reference to improve urban resilience, this paper introduces a spatial econometric model to analyze the influencing factors of urban resilience. The mean value of the urban comprehensive resilience value of the study area from 2006 to 2017 was selected as the explanatory variable of the spatial regression model, and the mean value of the regional-level city influence factor indicators from 2006 to 2017 was the explanatory variable. To more objectively analyze the data and obtain a reasonable scientific research result, this paper uses Excel to take the logarithm of all the variable values to eliminate the influence of the dimension [66].

#### 4.3.1. Selection of Influencing Factors

The city is a complex social system, and many factors affect urban resilience. Referring to the existing research results and considering the availability of data, this paper selected nine influencing factors from the four aspects of urban infrastructure, economy, ecology and society, as shown in Table 4. In terms of urban infrastructure, two indicators, the expenditure to maintain and build cities and per capita drainage pipe length, were selected. They represent the ability of a city to exhibit stiffness, strength and self-recovery in the event of “disturbance” and “shock” from natural disasters and emergencies [13,14].

In terms of urban economic factors, three indicators, the annual highway freight traffic, proportion of the actual use of foreign capital in GDP and GDP per square kilometer, were selected to discuss their impact on urban resilience. The annual highway freight traffic is an important manifestation of urban economic activities, but the excessive energy consumption and consumption of urban energy, resulting in increased levels of air pollutants and greenhouse gas emissions, will cause negative effects on urban environmental quality [72]. The proportion of the actual use of foreign capital in GDP is an important manifestation of the gradual integration of a country or regional economy into the regional production value chain. The excessive dependence of this indicator on the economic development of cities is not conducive to the management of urban economic risks, and it will also promote the expansion of economic activities, leading to massive consumption of natural resources. In the long run, it is not conducive to the improvement of urban resilience [73]. The GDP per square kilometer is the GDP value created by the city per square kilometer of land, and it is an important manifestation of the efficiency of urban land use [74]. In terms of urban social factors, we selected three indicators of the proportion of urban pension insurance coverage, proportion of the population with higher education, and urban population density to explore their impact on urban resilience. The increase in the proportion of urban pension insurance coverage can reduce the gap between the rich and poor in society and is an embodiment of the integration of urban residents and cities [75]. The proportion of the population with higher education not only has an important impact on the quality of life and satisfaction of urban residents but also increases the employment rate of cities and improves the overall learning ability of the city [76]. The urban population density is an important indicator reflecting the development and prosperity of a city and an important factor affecting the sustainable development of cities [77]. In terms of urban ecology, this paper selects the indicator of the carbon emissions per 10,000 CNY of GDP, which is an important manifestation of energy use efficiency and urban ecological environment [78].

#### 4.3.2. Estimation Results of the Ordinary Least Squares Model (OLS)

The results of the OLS model are shown in Table 5. Considering significance, the GDP per square kilometer, proportion of urban pension insurance coverage and proportion of the population with higher education passed the 1% significance test, and the annual highway freight traffic, carbon emissions per 10,000 CNY of GDP, and expenditure to maintain and build cities passed the 5% significance test. However, the proportion of the actual use of foreign capital in GDP, urban population density, and per capita drainage pipe length did not pass the significance test. The above variables are the main factors affecting the urban resilience of the study area, and the interpretation degree of resilience was 87.99%. From the positive and negative effects of the influencing factors, the annual highway freight traffic and carbon emissions per 10,000 CNY of GDP had negative impacts on urban resilience—that is, the greater their value, the lower the overall urban resilience—a finding in line with research expectations. The greater the GDP per square kilometer is, the higher the efficiency of urban land use is. Thus, under the circumstances of a certain area, the greater the GDP is per square kilometer, the higher the economic development degree and economic concentration of the region are, and the higher the economic resilience is of the city. The higher the proportion of urban pension insurance coverage is, the more robust the urban social security system is and the higher its social resilience is. The higher the proportion of the population with higher education is, the higher the importance of education in the city is, the higher the number of people receiving higher education is, and the better the education and culture of urban residents are, which can reduce unemployment and enhance urban social resilience. Therefore, the proportion of the population with higher education is also an important factor affecting the overall resilience of the city.

However, due to the spatial agglomeration characteristics of urban samples studied in urban resilience, urban resilience values show spatial autocorrelation and are not independent of each other, and do not conform to the assumption of the traditional OLS model [62]. Additionally, the diagnostic test of the estimated results of the OLS model (Table 6) revealed that the residual Moran’s I has a statistic of 2.9703, which passed the 5% significance level test (*p* = 0.0032), indicating a significant spatial dependence of the residual of the traditional OLS regression model. The *p* values of the Breusch‒Pagan test and Koenker‒Bassett test were 0.7352 and 0.3821, respectively, both of which were much greater than 0.05, indicating that the OLS regression model had heteroscedasticity. Therefore, spatial regression models need to be used that consider the effects of spatial dependence.

#### 4.3.3. Estimation Results of the Spatial Regression Model

Comparing the results of the spatial lag model (SLM), spatial error model (SEM) and ordinary least squares model (OLS) (Table 6 and Table 7), the correction R^2^ values for the SLM and SEM were 0.9066 and 0.9178, respectively, which were higher than those of the OLS. Using log-likelihood estimation to judge the model is not sufficient to compare the meaning of the adjusted R^2^. Therefore, the log likelihood function value can be used to judge the advantages and disadvantages of the spatial regression model and traditional OLS more scientifically. The log likelihood value of the SLM and SEM were 36.4814 and 35.9275, respectively, which are larger than those of OLS, and the AIC and SC values were −50.9628, −51.8550 and −28.6839, −31.6014, respectively, both of which were smaller than the AIC and SC values corresponding to the OLS model, indicating that the spatial measurement model is better than the traditional OLS model, and the fitting effect is better. Thus, based on the traditional OLS model, the spatial regression model should be introduced to explore the influence factors of urban resilience.

As previously described in the SLM and SEM, Lagrange multipliers (LM-lag and LM-error) and their robust LM diagnostics (robust LM-lag and robust LM-error) are used to determine which model is more realistic [67,79]. From the test results presented in Table 6, the *p* values corresponding to the statistics of LM-lag (0.0016) and LM-error (0.0000) passed the significance test, and LM-error was more significant than LM-lag. In addition, robust LM-error was significant at the 5% test level, while robust LM-lag failed to pass the significance test. According to Anselin’s discriminant criterion, SEM is more suitable to explore the influence factors of urban resilience [80].

Table 7 shows that the coefficients of the GDP per square kilometer, proportion of urban pension insurance coverage, proportion of the population with higher education, and expenditure to maintain and build cities passed the 1% significance test, and the coefficients of the proportion of the actual use of foreign capital in GDP and carbon emissions per 10,000 CNY of GDP passed the 5% significance test, and these influencing factors have obvious effects on urban resilience. However, the urban population density and per capita drainage pipe length did not pass the significance test, and the urban resilience effect of the study area was not significant. The positive and negative effects of the influencing factors are consistent with the reality—that is, except for the annual highway freight traffic, the proportion of the actual use of foreign capital in GDP and carbon emissions per 10,000 CNY of GDP on urban resilience, other influencing factors have positive correlations on urban resilience. This finding further clarifies that, if the annual highway freight traffic, proportion of the actual use of foreign capital in GDP or carbon emissions per 10,000 CNY of GDP of the city is greater, the urban resilience may be lower. However, the greater the values of the GDP are per square kilometer, proportion of urban pension insurance coverage, proportion of the population with higher education, and expenditure to maintain and build cities, the higher the resilience of urban development is.

The regression coefficient of each influencing factor is an important reflection of the influence of different factors on the degree of urban development resilience, and the greater the regression coefficient value is, the higher the degree of influence is. The absolute values of the regression coefficients of urban development resilience in Shaanxi, Henan, Anhui and Jiangsu provinces are ranked from the largest to the smallest as follows: proportion of urban pension insurance coverage, expenditure to maintain and build cities, proportion of the population with higher education, GDP per square kilometer, annual highway freight traffic, proportion of the actual use of foreign capital in GDP and carbon emissions per 10,000 CNY of GDP.

Specifically, the proportion of urban pension insurance coverage, expenditure to maintain and build cities, proportion of the population with higher education and GDP per square kilometer increased by 1%, and the levels of urban development resilience increased by 0.356%, 0.073%, 0.068% and 0.058%, respectively. The annual highway freight traffic, proportion of the actual use of foreign capital in GDP and carbon emissions per 10,000 CNY of GDP increased by 1%, and the urban development resilience levels decreased by 0.043%, 0.022% and 0.008%, respectively. The proportion of urban pension insurance coverage has the greatest impact on the urban development resilience of the study area, mainly because, with continuous improvement of the level of urbanization development, the phenomenon of population aging is becoming increasingly serious, and urban pension insurance has become an important issue facing the sustainable development of cities and development of healthy cities. Therefore, improving urban pension insurance coverage is an important direction to improve urban development resilience.

The impact of the expenditure to maintain and build cities and proportion of the population with higher education on urban development resilience are also prominent. The expenditure to maintain and build cities is an important source of funds to improve urban infrastructure construction, and the proportion of the population with higher education is an important indicator of the level of urban education development. Increasing the expenditure to maintain and build cities to solve the problem of urban infrastructure lag and attaching importance to the development of education can improve the overall quality and employment rate of urban citizens, both of which can improve urban resilience to some extent. The impact of the GDP per square kilometer on urban resilience is relatively lagging, and its impact is not as strong as we expected. Additionally, the regression coefficient only passed the 10% significance test, possibly due to the traditional sense that GDP per square kilometer is an important indicator to measure a city’s economic level, which leads to the mistaken belief that GDP per square kilometer has a significant impact on urban resilience. Moreover, in fact, urban resilience should be considered as being caused by the joint effect of other factors.

However, the impact of the proportion of the actual use of foreign capital in GDP and carbon emissions per 10,000 CNY of GDP on urban resilience is negative and weak, indicating that the importance of these two aspects is not sufficient in the process of urbanization development in China, which is also an important link that needs to be strengthened to enhance urban development resilience. Particularly, in the process of urban development, the ecological and environmental problems of cities are receiving increasing attention, and the reduction of carbon emissions can contribute to optimization of the ecological environment, which can improve the urban environmental quality to a certain extent and provide important support for urban resilience.

## 5. Conclusions and Recommendations

### 5.1. Conclusions

The essence of urban resilience research is to actively explore adaptive adjustment methods and approaches for the uncertainty disturbances faced by modern cities. Strengthening urban resilience is an important part of promoting urban development from scale expansion to quality improvement. This paper presented a study of 56 prefecture-level cities in Shaanxi, Henan, Anhui, and Jiangsu provinces from 2006 to 2017:

(1) During the study period, the urban resilience showed different degrees of improvement; overall, an upward trend was observed. The urban resilience value ranged between 0.22 and 0.42, which was relatively small, indicating that the urban resilience of the study area warrants improvement.

(2) The Moran index of urban resilience showed an overall upward trend of fluctuations, indicating a significant positive correlation in the spatial distribution of urban resilience in the study area, the agglomeration characteristics are significant, and the spatial autocorrelation of urban resilience has a further strengthening trend on the time scale.

(3) The spatial agglomeration characteristics of urban resilience in Eastern, Central and Western China were obvious, and the distribution of “cold hot spots” was not balanced. Compared with that of 2006, the scope of the “H-H” cluster area in 2017 was further expanded. In 2017, the “L-L” cluster area also experienced major expansion and local regional contraction changes. The scope of the “L-H” cluster was narrowed and only appeared in a few cities. The “H-L” cluster area also appeared in the study area in 2017. During the period, policies such as “Western Development” and “Rise of Central China” played an important role in promoting the change of urban resilience.

(4) In terms of factors affecting urban resilience, the proportion of the actual use of foreign capital in GDP and carbon emissions per 10,000 CNY of GDP have negative impacts, and the GDP per square kilometer, proportion of urban pension insurance coverage, proportion of the population with higher education, and expenditure to maintain and build cities have positive impacts. The degree of influence is ranked from the highest to the lowest as follows: the proportion of urban pension insurance coverage, expenditure to maintain and build cities, proportion of the population with higher education, GDP per square kilometer, annual highway freight traffic, proportion of the actual use of foreign capital in GDP and carbon emissions per 10,000 CNY of GDP.

### 5.2. Recommendations

It is important to clarify the spatial and temporal differentiation patterns of urban resilience and their influencing factors to enhance urban risk adaptability and the urban sustainable development potential. The ESDA was used to explore the spatial characteristics of urban resilience under spatial interaction, and the spatial econometric model was also used to analyze the mechanism of the influence of urban resilience impact factors in this paper. The urban development process is greatly influenced by many other factors, but this paper only selected 29 indicators to analyze and evaluate the urban resilience of the study area. The incompleteness of socio-economic data in individual cities will also have a greater impact on urban resilience. Therefore, the results of the study may be inconsistent with the actual situation in the study area. It is necessary to consider as many of the indicators as possible in future research, and it can portray urban resilience more deeply and comprehensively. According to the evaluation of urban resilience and the analysis of influencing factors in the study area, it is revealed that the improvement of urban resilience can be strengthened from the following aspects:

(1) The resilience city construction and development planning strategy should include its infrastructure development plan. It is necessary to vigorously promote the improvement of the ability of infrastructure to keep pace with the times and the level of intelligence and to enhance the resilience of urban disasters, so as to further enhance the resilience of urban infrastructure.

(2) The urban economy should focus on its own diversity, instead of relying too much on foreign investment. Establishing an economical structural system of resilient cities and an effective mechanism for economic development can effectively promote the transformation of the urban economic development mode, and it is an important force to enhance urban economic resilience in all directions.

(3) In the planning and construction process of urban resilience, we should pay attention to the multiple benefits and function exploration of the ecological environment and strengthen the management of ecosystems, incorporate the ecological environment optimization policy into the planning of resilient urban construction, further reduce the probability of urban disasters, and effectively promote urban ecological resilience.

(4) We should attach importance to strengthening the social public policy coordination and integration chain and actively establish a resilience system evaluation and implementation mechanism. This not only helps to maximize the rational use and development of social resources in the process of urban development, but also effectively promotes urban social resilience.

(5) It is necessary to continue to deepen the understanding of the basic connotation and intrinsic properties of resilient cities and conduct multi-dimensional analysis. We also need to further improve the top-level design of resilient urban development and rationally determine the strategic position of resilient cities, so as to formulate scientific and rational action plans.

## Figures and Tables

**Table 1:** Weight and assessment indicator system of urban resilience.

Level 1 Indicator	Weight	Level 2 Indicator	Weight	Unit	Reference
Urban infrastructure resilience	0.3287	Number of internet users per 100 people	0.0575	Household	[46,47,48]
		Number of health care beds per 10,000 people	0.0286	Bed	[46,50,51]
		Number of public transportation vehicles per 10,000 people	0.0399	Unit	[47,49]
		Per capita area of paved roads in city	0.0330	m²	[49,51]
		Per capita annual electricity consumption	0.0342	kW·h/person	[50,51]
		Per capita postal expenditure	0.0839	CNY	[48,50]
		Number of mobile phones per 100 people	0.0516	Household	[47,49]
Urban economic resilience	0.3185	Fiscal deficit rate	0.0113	%	[49,51]
		Proportion of private and individual employment in urban areas to the number of employed people in the city	0.0286	%	[46,48]
		Proportion of GDP increased by the tertiary industry	0.0242	%	[46,50]
		Financial interrelations ratio	0.0501	m²	[47,49,50]
		Industrial structure diversification index	0.0345	—	[48,50]
		Proportion of financial expenditure on science and technology	0.0471	%	[47,49,50]
		Per capita GDP	0.0539	CNY	[46,47,48]
		Per capita retail sales amount of consumer goods	0.0688	CNY	[47,50,51]
Urban ecological resilience	0.1048	Electricity consumption per 10,000 CNY of GDP	0.0049	kW·h/10,000 CNY	[50,51]
		Volume of sulfur dioxide emissions	0.0064	Ton	[46,48]
		Volume of industrial waste water discharged	0.0042	10 000 tons	[46,47]
		Volume of industrial soot (dust) emissions	0.0064	Ton	[46,48]
		Green coverage rate in urban constructed areas	0.0101	%	[46,47,50]
		Per capita area of parks and green land	0.0652	m²	[46,47,48,49]
		Ratio of industrial solid wastes comprehensively utilized	0.0076	%	[46,47,48]
Urban social resilience	0.0248	Number of doctors per 10,000 people	0.0228	Person	[46,49]
		Proportion of employees in public administration and social organizations	0.0290	%	[46,51]
		Proportion of education and financial expenditure	0.0156	%	[50,51]
		Collections of public libraries per 100 persons	0.0700	Piece	[46,49]
		Per capita household deposit balance	0.0715	CNY	[46,48,50]
		Proportion of unemployment in urban areas	0.0077	%	[46,50,51]
		Average wage of employed staff and workers	0.0314	CNY	[46,49,50]

**Table 2:** The comprehensive evaluation results of urban resilience in provinces from 2006 to 2017.

Year	Shaanxi Province	Henan Province	Anhui Province	Jiangsu Province
2006	0.2344	0.2069	0.2341	0.4007
2007	0.2427	0.2100	0.2484	0.4257
2008	0.2249	0.2068	0.2387	0.3957
2009	0.2344	0.2175	0.2440	0.3840
2010	0.2378	0.2239	0.2525	0.3939
2011	0.2515	0.2265	0.2634	0.4327
2012	0.2406	0.2176	0.2560	0.4236
2013	0.2350	0.2225	0.2416	0.3963
2014	0.2346	0.2305	0.2454	0.4269
2015	0.2453	0.2340	0.2519	0.4224
2016	0.2427	0.2212	0.2408	0.4095
2017	0.2575	0.2308	0.2619	0.4024
Average value	0.2401	0.2207	0.2482	0.4095
Changes in urban resilience from 2006 to 2017	0.0231	0.0239	0.0278	0.0017
Rate of change in 2017	8.97%	10.36%	10.61%	0.42%

**Table 3:** The Moran’s index and *p* values of the urban resilience from 2006 to 2017.

Year	Moran’s I	*p*	Z	Year	Moran’s I	*p*	Z
2006	0.3560	0.001	4.3897	2012	0.3820	0.001	4.7587
2007	0.3452	0.001	4.2475	2013	0.3911	0.001	4.9174
2008	0.3880	0.001	4.7378	2014	0.3419	0.001	4.2849
2009	0.3559	0.001	4.4188	2015	0.3524	0.001	4.4715
2010	0.3718	0.001	4.6655	2016	0.3621	0.001	4.5364
2011	0.4345	0.001	5.3824	2017	0.3751	0.001	4.6752

**Table 4:** Description of influencing factors.

Indicator Name	Unit	Data Sources
Annual highway freight traffic	10,000 tons	China City Statistical Yearbook (2006–2017)
Urban population density	Person/km^2^	China City Statistical Yearbook (2006–2017)
Proportion of actual use of foreign capital in GDP	%	China City Statistical Yearbook (2006–2017)
GDP per square kilometers	CNY/km²	China City Statistical Yearbook (2006–2017)
Expenditure to maintain and build cities	10,000 CNY	China City Statistical Yearbook (2006–2017)
Per capita drainage pipe length	m	China City Statistical Yearbook (2006–2017)
Carbon emissions per 10,000 CNY of GDP	Ton/10,000 CNY	Statistical yearbooks of provinces and municipalities
Proportion of the population with higher education	%	China City Statistical Yearbook (2006–2017)
Proportion of urban pension insurance coverage	%	Statistical yearbooks of provinces and municipalities

**Table 5:** Estimation results of the ordinary least squares model (OLS).

Variable	Coefficient	Standard Deviation	T Statistic	*p* Value
Annual highway freight traffic	−0.0449 **	0.0415	−3.0838	0.0162
Proportion of actual use of foreign capital in GDP	−0.0191	0.0196	−0.9747	0.3348
GDP per square kilometers	0.0429 ***	0.0438	4.9800	0.0012
Urban population density	0.0167	0.0306	0.5462	0.58755
Proportion of urban pension insurance coverage	0.3974 ***	0.0530	7.5014	0.0000
Proportion of the population with higher education	0.0607 ***	0.0313	3.9382	0.0088
Carbon emissions per 10,000 CNY of GDP	−0.0070 **	0.0347	−2.9513	0.0140
Expenditure to maintain and build cities	0.0691 **	0.0213	2.7636	0.0221
Per capita drainage pipe length	0.0137	0.0293	0.4680	0.2420
R^2^	0.8995			
Adjusted R^2^	0.8799			
Log likelihood	34.6374			
AIC	−49.2747			
SC	−29.0212			

Note: ** and *** indicate the significance levels are 0.05 and 0.01 respectively.

**Table 6:** Diagnostics for the OLS model.

Test Statistics	Statistics	*p* Value
Breusch‒Pagan	6.0470	0.7352
Koenker‒Bassett	9.6207	0.3821
Moran’s I (error)	2.9703	0.0032
LM-lag	13.4226	0.0016
Robust LM-lag	3.2507	0.7348
LM-error	11.2867	0.0000
Robust LM-error	0.1148	0.0029

**Table 7:** The estimation results of the spatial lag model and spatial error model.

Variable	SLM			SEM		
	Coefficient	Standard Deviation	*p* Value	Coefficient	Standard Deviation	*p* Value
Annual highway freight traffic	−0.0430 ***	0.0363	0.0051	−0.0268 ***	0.0341	0.0018
Proportion of actual use of foreign capital in GDP	−0.0110 **	0.0176	0.0305	−0.0216 **	0.0184	0.0393
GDP per square kilometers	0.0426 *	0.0383	0.0563	0.0582 *	0.0399	0.0526
Urban population density	0.0262	0.0271	0.3334	0.0249	0.0258	0.3356
Proportion of urban pension insurance coverage	0.3462 ***	0.0523	0.0000	0.3556 ***	0.0519	0.0000
Proportion of the population with higher education	0.0740 ***	0.0281	0.0084	0.0684 ***	0.0281	0.0148
Carbon emissions per 10,000 CNY of GDP	−0.0143 **	0.0321	0.0351	−0.0077 **	0.0319	0.0286
Expenditure to maintain and build cities	0.0740 ***	0.0187	0.0001	0.0726 ***	0.0185	0.0001
Per capita drainage pipe length	0.0230	0.0259	0.3758	0.0220	0.0253	0.3846
R^2^	0.9066			0.9178		
Log likelihood	35.9275			36.4814		
AIC	−50.9628			−51.8550		
SC	−28.6839			−31.6014		

Note: *, ** and *** indicate the significance level is 0.1, 0.05 and 0.01 respectively.

## References

[1] Fan Y., Yu G.M., He Z. (2017). Origin, spatial pattern, and evolution of urban system: Testing a hypothesis of “urban tree”. Habitat Int..

[2] Spaans M., Waterhout B. (2017). Building up resilience in cities worldwide—Rotterdam as participant in the 100 Resilient Cities Programme. Cities.

[3] Li T.Y., Niu P.Y., Gu C.L. (2015). A review on research frameworks of resilient cities. Urban Plan. Forum.

[4] Chen M.X., Liu W.D., Tao X.L. (2013). Evolution and assessment on China’s urbanization 1960–2010: Under-urbanization or over-urbanization?. Habitat Int..

[5] Yang J., Wu T.H., Gong P. (2017). Implementation of China’s new urbanization strategy requires new thinking. Chin. Sci. Bull..

[6] Tao Y., Li F., Crittenden J.C., Lu Z., Sun X. (2016). Environmental Impacts of China’s Urbanization from 2000 to 2010 and Management Implications. Environ. Manag..

[7] Yang K., Yu Z.Y., Luo Y., Zhou X.L., Shang C.X. (2019). Spatial-Temporal Variation of Lake Surface Water Temperature and Its Driving Factors in Yunnan-Guizhou Plateau. Water Resour. Res..

[8] Huang X., Schneider A., Friedl M.A. (2016). Mapping sub-pixel urban expansion in China using MODIS and DMSP/OLS nighttime lights. Remote Sens. Environ..

[9] Luo Y., Li Q.L., Yang K., Xie W.Q., Zhou X.L., Shang C.X., Xu Y.T., Zhang Y., Zhang C. (2019). Thermodynamic analysis of air-ground and water-ground energy exchange process in urban space at micro scale. Sci. Total Environ..

[10] Liu F., Zhang Z.X., Wang X. (2016). Forms of urban expansion of Chinese municipalities and provincial capitals, 1970s–2013. Remote Sens..

[11] Yang K., Pan M., Luo Y., Chen K.X., Zhao Y.S., Zhou X.L. (2019). A time-series analysis of urbanization-induced impervious surface area extent in the Dianchi Lake watershed from 1988–2017. Int. J. Remote Sens..

[12] Meerow S., Newell J.P., Stults M. (2016). Defining urban resilience: A review. Landsc. Urban Plan..

[13] Li T.Y. (2017). New Progress in Study on Resilient Cities. Urban Plan. Int..

[14] Shao Y.W., Xu J. (2015). Understanding Urban Resilience: A Conceptual Analysis Based on Integrated International Literature Review. Urban Plan. Int..

[15] Davoudi S., Resilience A. (2012). A bridging concept or a dead end?. Plan. Theory Pract..

[16] Holling C.S. (1973). Resilience and stability of ecological systems. Annu. Rev. Ecol. Syst..

[17] Meerow S., Newell J.P. (2019). Urban resilience for whom, what, when, where, and why?. Urban Geogr..

[18] Gunderson L.H., Holling C.S. (2001). Panarchy: Understanding Transformations in Human and Natural Systems.

[19] Wildavsky A.B. (1988). Searching for Safety.

[20] Leichenko R. (2011). Climate change and urban resilience. Curr. Opin. Environ. Sustain..

[21] Adger W.N. (2000). Social and ecological resilience: Are they related?. Prog. Hum. Geogr..

[22] Paton D., Johnston D. (2001). Disasters and communities: Vulnerability, resilience and preparedness. Dis. Prev. Manag..

[23] Rose A., Lim D. (2002). Business interruption losses from natural hazards: Conceptual and methodological issues in the case of the Northridge earthquake. Environ. Hazards.

[24] Polèse M. (2010). The Resilient City: On the Determinants of Successful Urban Economies.

[25] Adger W.N. (2006). Vulnerability. Glob. Environ. Chang..

[26] Ernstson H., Van der Leeuw S.E., Redman C.L., Meffert D.J., Davis G., Alfsen C., Elmqvist T. (2010). Urban transitions: On urban resilience and human-dominated ecosystems. Ambio.

[27] Liu Z.M., Xiu C.L., Song W. (2019). Landscape-Based Assessment of Urban Resilience and Its Evolution: A Case Study of the Central City of Shenyang. Sustainability.

[28] Zhang C., Li Y.F., Zhu X.D. (2016). A Social-Ecological Resilience Assessment and Governance Guide for Urbanization Processes in East China. Sustainability.

[29] Wang Z., Deng X.Z., Wong C., Li Z.H., Chen J.C. (2018). Learning urban resilience from a social-economic-ecological system perspective: A case study of Beijing from 1978 to 2015. J. Clean. Prod..

[30] Xie X.F., Pu L.J. (2017). Assessment of urban ecosystem health based on matter element analysis: A case study of 13 cities in Jiangsu Province, China. Int. J. Environ. Res. Public Health.

[31] Wang L., Xue X.L., Wang Z.Y., Zhang L.S. (2018). A unified assessment approach for urban infrastructure sustainability and resilience. Adv. Civ. Eng..

[32] Li M., Wang H.W., Wang H.S. (2019). Resilience Assessment and Optimization for Urban Rail Transit Networks: A Case Study of Beijing Subway Network. IEEE Access.

[33] Fang D., Li Z., Li N., Han L., Wu J., Lu X., Kong X., Li Y., Lu X. (2017). Urban resilience: A perspective of system of systems in trio spaces. China Civ. Eng. J..

[34] Xie Q.H. (2017). Enlightenment of Resilient City Construction Policy in Developed Country. Sci. Decis. Mak..

[35] He B.J., Zhu J., Zhao D.X., Gou Z.H., Qi J.D., Wang J.S. (2019). Co-benefits approach: Opportunities for implementing sponge city and urban heat island mitigation. Land Use Policy.

[36] He B.J. (2019). Towards the next generation of green building for urban heat island mitigation: Zero UHI impact building. Sustain. Cities Soc..

[37] Ma L.B., Chen M.M., Fang F., Che X.L. (2019). Research on the spatiotemporal variation of rural-urban transformation in underdeveloped regions and its driving mechanisms: Gansu Province in western China as an example. Sustain. Cities Soc..

[38] Adedeji T., Proverbs D., Xiao H., Cobbing P., Oladokun V. (2019). Making Birmingham a Flood Resilient City: Challenges and Opportunities. Water.

[39] Mierzejewska L., Magdalena W. (2018). City resilience vs. resilient city: Terminological intricacies and concept inaccuracies. Quaest. Geogr..

[40] Jabareen Y. (2013). Planning the resilient city: Concepts and strategies for coping with climate change and environmental risk. Cities.

[41] The People’s Government of Shaanxi Provincial. http://dfz.shaanxi.gov.cn/sxsq/201610/t20161020_679566.html.

[42] The People’s Government of Henan Provincial. https://www.henan.gov.cn/2018/05-31/2408.html.

[43] The People’s Government of Anhui Provincial. http://www.ah.gov.cn/UserData/SortHtml/1/8394315416.html.

[44] The People’s Government of Jiangsu Provincial. http://www.jiangsu.gov.cn/col/col31359/index.html.

[45] Chen W., Shen Y., Wang Y. (2018). Evaluation of economic transformation and upgrading of resource-based cities in Shaanxi province based on an improved TOPSIS method. Sustain. Cities Soc..

[46] Fang C.L., Wang Y., Fang J.W. (2015). A comprehensive assessment of urban vulnerability and its spatial differentiation in China. Acta Geogr. Sin..

[47] Sun Y., Zhang L.C., Yao S.M. (2017). Evaluating resilience of prefecture cities in the Yangtze River delta region from a socio-ecological perspective. China Popul. Resour. Environ..

[48] Li T., Gu C.L. Research on the Index System of Resilient City in China. Proceedings of the 2014 2nd International Conference on Social Sciences Research.

[49] He B.J., Zhao D.X., Zhu J., Darko A., Gou Z.H. (2018). Promoting and implementing urban sustainability in China: An integration of sustainable initiatives at different urban scales. Habitat Int..

[50] Bozza A., Asprone D., Manfredi G. (2015). Developing an integrated framework to quantify resilience of urban systems against disasters. Nat. Hazards.

[51] Bănică A., Muntele I. (2015). Urban vulnerability and resilience in post-communist Romania (comparative case studies of Iași and Bacău cities and metropolitan areas). Carpath. J. Earth Environ. Sci..

[52] McPhearson T., Haase D., Kabisch N., Gren A. (2016). Advancing understanding of the complex nature of urban systems. Ecol. Ind..

[53] Lin W.Y., Hung C.T. (2016). Applying spatial clustering analysis to a township-level social vulnerability assessment in Taiwan. Geomat. Nat. Hazards Risk.

[54] Zhang B., Zhang Y., Chen D., White R.E., Li Y. (2004). A quantitative evaluation system of soil productivity for intensive agriculture in China. Geoderma.

[55] Xu Z., Zhao N. (2016). Information fusion for intuitionistic fuzzy decision making: An overview. Inf. Fusion.

[56] Peeters L., Chasco C. (2006). Ecological inference and spatial heterogeneity: An entropy-based distributionally weighted regression approach. Pap. Reg. Sci..

[57] Siahkamari S., Haghizadeh A., Zeinivand H., Tahmasebipour N., Rahmati O. (2018). Spatial prediction of flood-susceptible areas using frequency ratio and maximum entropy models. Geocarto Int..

[58] Zhang X.Q. (2016). The trends, promises and challenges of urbanisation in the world. Habitat Int..

[59] Han Z.L., Liu T.B. (2009). Analysis of the characteristics and spatial differences of urbanization quality of cities at prefecture level and above in China. Geogr. Res..

[60] Shen L.Y., Shuai C.Y., Jiao L.D., Tan Y.T., Song X.N. (2017). Dynamic sustainability performance during urbanization process between BRICS countries. Habitat Int..

[61] Griffith D.A. (2011). Visualizing analytical spatial autocorrelation components latent in spatial interaction data: An eigenvector spatial filter approach. Comput. Environ. Urban.

[62] Zhang X.S., Zhang M.M., He J., Wang Q.X., Li D.S. (2019). The Spatial-Temporal Characteristics of Cultivated Land and Its Influential Factors in The Low Hilly Region: A Case Study of Lishan Town, Hubei Province, China. Sustainability.

[63] Oliveira D.G., Diniz-Filho J.A.F. (2010). Spatial patterns of terrestrial vertebrate richness in Brazilian semiarid, Northeastern Brazil: Selecting hypotheses and revealing constraints. J. Arid Environ..

[64] Kawaguchi D., Yukutake N. (2017). Estimating the residential land damage of the Fukushima kernel accident. J. Urban Econ..

[65] Mahara G., Wang C., Yang K., Chen S., Guo J., Gao Q., Wang W., Wang Q., Guo X. (2016). The Association between Environmental Factors and Scarlet Fever Incidence in Beijing Region: Using GIS and Spatial Regression Models. Int. J. Environ. Res. Public Health.

[66] Yang X.Y., Jin W. (2010). GIS-based spatial regression and prediction of water quality in river networks: A case study in Iowa. J. Environ. Manag..

[67] Egger P., Pfaffermayr M. (2016). A generalized spatial error components model for gravity equations. Empir. Econ..

[68] Dai Z., Guldmann J.M., Hu Y.F. (2018). Spatial regression models of park and land-use impacts on the urban heat island in central Beijing. Sci. Total Environ..

[69] Anselin L. (2003). Spatial Externalities, Spatial Multipliers, and Spatial Econometrics. Int. Reg. Sci. Rev..

[70] Li Y.F., Li D. (2014). Assessment and forecast of Beijing and Shanghai’s urban ecosystem health. Sci. Total Environ..

[71] Chun B., Guldmann J.M. (2014). Spatial statistical analysis and simulation of the urban heat island in high-density central cities. Landsc. Urban Plan..

[72] Hao H., Geng Y., Li W.Q., Guo B. (2015). Energy consumption and GHG emissions from China’s freight transport sector: Scenarios through 2050. Energy Policy.

[73] Liargovas P.G., Skandalis K.S. (2012). Foreign direct investment and trade openness: The case of developing economies. Soc. Indic. Res..

[74] Hua W., Fan L., Wu Q., Peng B. (2005). Regression analysis of urban land value influencing factors: For Jiangsu province. Econ. Geogr..

[75] Barbier E.B. (2010). Poverty, development, and environment. Environ. Dev. Econ..

[76] Winters J.V. (2011). Human capital, higher education institutions, and quality of life. Reg. Sci. Urban Econ..

[77] Cole M.A., Neumayer E. (2004). Examining the impact of demographic factors on air pollution. Popul. Environ..

[78] Lin B.Q., Lin X.Y. (2009). China’s Carbon Dioxide Emissions under the Urbanization Process: Influence Factors and Abatement Policies. Econ. Res. J..

[79] Anselin L., Florax R., Rey S.J. (2004). Advances in Spatial Econometrics: Methodology, Tools and Applications.

[80] Anselin L., Bera A., Ullah A., Giles D. (1998). Spatial Dependence in Linear Regression Models with an Introduction to Spatial Econometrics. Handbook of Applied Economic Statistics.

